# How you perceive threat determines your behavior

**DOI:** 10.3389/fnhum.2013.00632

**Published:** 2013-10-08

**Authors:** Orlando Fernandes, Liana C. L. Portugal, Rita C. S. Alves, Rafaela R. Campagnoli, Izabela Mocaiber, Isabel P. A. David, Fátima C. S. Erthal, Eliane Volchan, Leticia de Oliveira, Mirtes G. Pereira

**Affiliations:** ^1^Department of Physiology and Pharmacology, Laboratory of Neurophysiology of Behavior, Biomedical Institute, Federal Fluminense UniversityNiterói, RJ, Brazil; ^2^Laboratory of Neurobiology II, Institute of Biophysics Carlos Chagas Filho, Federal University of Rio de JaneiroRio de Janeiro, RJ, Brazil

**Keywords:** emotion, attention, reaction time, threat stimuli, defensive responses, behavior

## Abstract

The prioritization of processing emotional stimuli usually produces deleterious effects on task performance when it distracts from a task. One common explanation is that brain resources are consumed by emotional stimuli, diverting resources away from executing the task. Viewing unpleasant stimuli also generates defensive reactions, and these responses may be at least partially responsible for the effect of the emotional modulation observed in various reaction time (RT) paradigms. We investigated whether modulatory effects on RT vary if we presented threat stimuli to prompt different defensive responses. To trigger different responses, we manipulated threat perception by moving the direction of threatening stimuli. Threatening or neutral stimuli were presented as distractors during a bar orientation discrimination task. The results demonstrated that threat stimuli directed toward the observer produced a decrease in RT; in contrast, threat stimuli directed away from the observer produced an increase in RT, when compared to neutral stimuli. Accelerated RT during directed toward threat stimuli was attributed to increased motor preparation resulting from strong activation of the defense response cascade. In contrast, directed away threat stimuli likely activated the defense cascade, but less intensively, prompting immobility. Different threat stimuli produced varying effects, which was interpreted as evidence that the modulation of RT by emotional stimuli represents the summation of attentional and motivational effects. Additionally, participants who had been previously exposed to diverse types of violent crime were more strongly influenced by threat stimuli directed toward the observer. In sum, our data support the concept that emotions are indeed action tendencies.

## Introduction

There is considerable evidence suggesting that emotion affects behavior; understanding the ways that processing emotional visual stimuli produces this effect is of great interest. Processing emotional stimuli is usually prioritized relative to neutral stimuli (Öhman et al., 2001; Ishai et al., 2004), although several factors, including attention, cognitive regulation and individual traits, may modulate the extent of affective stimuli processing that occurs (Ochsner and Gross, 2005; Pessoa, 2005; Souza et al., 2007; Oliveira et al., 2009; Mocaiber et al., 2010, 2011; Pessoa, 2010; Menezes et al., 2012). Prioritization of emotional stimuli processing may induce deleterious effects on task performance, e.g., when processing emotional stimuli is irrelevant to the task at hand (Hartikainen et al., 2000; Tipples and Sharma, 2000). For example, the presence of a central unpleasant picture may increase reaction times (RTs) when participants complete a peripheral bar orientation discrimination task (Erthal et al., 2005). The effects of emotional stimuli on task performance are commonly thought to be mediated by attention (Pessoa et al., 2002; Pourtois et al., 2006) with the reasoning that prioritizing emotional items diverts resources away from processing neutral items, which slows RT.

The attentional effects of emotional stimuli have been widely reported; additionally, viewing unpleasant stimuli generates defensive reactions (Bradley et al., 2001; Azevedo et al., 2005). Considering an evolutionary perspective of emotion, animals must be able to identify threat signals and act on them effectively to avoid body envelope violation and increase the chance of survival. In fact, Darwin (1872) argued that emotions are adaptive insofar as they prompt actions that are beneficial to the organism. In this theoretical framework, it is expected that processing emotional items should also influence motor output by preparing individuals for action. Many neuroimaging studies support the idea that activity in various motor-related areas is modulated by emotional processing in humans (Anderson and Phelps, 2001; Oliveri et al., 2003; Baumgartner et al., 2007; Butler et al., 2007; Hajcak et al., 2007; de Oliveira et al., 2012). In addition, it is reasonable to expect an interaction between attention/perceptual processes and motivational processes during defense. In fact, Lang et al. (1997) suggests heightened perceptual processing in the context of defensive response. According to this view, motivational circuits are triggered by external (environment) or internal (memory) cues facilitating cognitive processes that enhance perception in order to select an appropriate action. For instance, a recent study showed that fear facilitates visual perception of external cues (Keil et al., 2010). Therefore, defense responses triggered during aversive contexts seems to produce important modulation on visual perception as well as on motor output.

Previous studies conducted by our group investigated whether defensive responses that are prompted by unpleasant stimuli are at least partially responsible for emotional modulatory effects on RT. Pereira et al. (2004, 2006) showed that viewing pictures of mutilated bodies increased RTs in performing a simple non-emotional visual detection task. This interference effect was accompanied by enhanced activity of the entire circuit involved in the task when presenting mutilated bodies compared to neutral pictures (Pereira et al., 2010). The increase in RT was interpreted as being the result of the instatement of a defensive emotional state, likely an immobility reaction, and increased activity of the motor circuit was attributed to preparing for a motor response in this aversive context (Pereira et al., 2010). Slower RTs to perform a RT task during the visualization of blood/injury pictures were also described by Buodo et al. (2002).

The stimuli used in the experiments cited above were pictures of mutilation and injured individuals; these images may signal that a potential life threat is present in the environment. In fact, Azevedo et al. (2005) reported direct evidence that mutilation pictures induce a defensive freezing-like response in humans. Freezing responses, or “attentive immobility” as described by Marks (1987), constitute a common adaptive defensive behavior when complex animals confront a potential threat (Blanchard et al., 1986; Kalin, 1993). However, the freezing reaction is just one of the possible responses implemented by the defense system. Animal behavioral studies have explored the defensive system thoroughly and report that there are many different reactions to threat (e.g., Ratner, 1967; Bolles, 1970; Blanchard and Blanchard, 1989; Fanselow, 1991, 1994). A very influential model that emerged from this literature is the “predator stage model” (for a review, see Lang et al., 2000). Lang et al. (1997) proposed an adaptation of this model to explain human reactions that have been observed in the laboratory when participants view unpleasant stimuli. The main idea behind their model is that as threat levels increase, defensive response strategies vary and increase systematically, changing from passive freezing to active flight, or attack if escape is not possible. Thus, considering this model, aversive pictures with differing levels of threat intensity may produce opposing influences on motor output, e.g., immobility versus overt defensive action.

Typically, healthy participants exhibit freezing in response to viewing aversive pictures that are presented in the laboratory (Cuthbert et al., 1996; Bradley et al., 2001). However, indications that participant responses approach overt defensive action when exposed to aversive pictures are scarce and have been described in phobic participants when viewing pictures of their phobic object (Hamm et al., 1997; Klorman et al., 1977; Wendt et al., 2008). It is possible that multiple characteristics of stimuli generate the range of behaviors that might be triggered in reaction to aversive stimuli. Blanchard et al. (2001) demonstrated that specific features of threatening stimuli that are determinants of the defensive strategy triggered in rodents appear to be equally fundamental in humans. According to these authors, these features include the threat magnitude, the escapability of the situation, the distance between the threat and the subject and the presence of available hiding places. For example, the perception of a predator attack may evoke a flight response in the presence of an available escape route; however, freezing may be exhibited if escape is not possible, and the likelihood of defensive attack behavior increases as the threat approaches and inescapability increases. Therefore, it is reasonable to suspect that using aversive pictures that are perceived as intense, imminent and inescapable threats may prompt overt defensive actions in the laboratory, such as a defensive attack.

One aspect of threat stimuli that may increase the perception of threat imminence is the direction of threat, i.e., whether the threat is directed toward or away from the observer. Flykt et al. (2007) tested the effect of threat direction in conditioning experiments using conditioned biological threats (e.g., snakes) or cultural threats (e.g., guns). The results revealed that threatening stimuli directed toward the observer produced conditioned skin conductance responses that were resistant to backward masking, regardless of whether the threat was due to biological or cultural causal factors. Threat stimuli directed away from the observer produced conditioned skin conductance responses, but backward masking abolished this effect. The authors emphasized that the direction of the threat stimulus was the critical factor modulating defensive responses and that threat stimuli directed toward the observer increased threat imminence, which enhanced psychophysiological responses. Hugdahl and Johnsen (1989) have also previously studied the importance of threat direction. These authors have demonstrated that observers exhibit stronger resistance to extinction for a gun pointed toward them compared to a gun pointed away from them. Similarly, Dimberg and Öhman (1983) reported that angry faces were more effective as conditioned stimuli only if they were directed toward the observer. In a recent and very interesting paper, Grèzes et al. (2013) found that “self-directed body expressions” of anger (those directed toward the observer) triggered higher corrugator reactivity compared to “other-directed bodies” (directed away from the observer). In addition, according to the participant’s appraisal of stimuli, the perception of self-directed anger expressions produced greater feelings of threat compared to those associated with other-directed body expressions.

In the present study, we investigated whether the emotional modulation of behavior typically produced by aversive pictures could vary by manipulating the perception of the magnitude, imminence and inescapability of threat stimuli. The direction of threat stimuli was the key factor that was manipulated to attempt to activate different defensive responses, which are meant to represent different positions along the threat imminence continuum. Threat stimuli directed toward the observer (e.g., guns) were expected to produce more intense activations of the defensive cascade than threat stimuli directed away from the observer. Threat or neutral stimuli were presented as distractors while participants were asked to perform a bar orientation discrimination task. The type of modulation typically produced by unpleasant stimuli in this paradigm is an interference effect (e.g., Erthal et al., 2005), i.e., the slowing down of RT. This effect is commonly described as the result of brain resources being consumed by emotional stimuli. Our hypothesis was that presenting threat stimuli that activate the defense cascade more powerfully and thus prompting overt defensive actions may abolish or supplant the interference commonly produced by attentional effects. If this hypothesis is true, RTs associated with threat stimuli should be equal to or shorter than RTs for neutral trials. However, for threat stimuli that are perceived as farther away from the observer, the expected defensive response would likely be an immobility reaction. If this were the case, the attentional effects and motor system modulation should produce increased RTs in threat trials when compared to neutral trials. Considering that threat stimuli were only used as distractors and that the task remained the same throughout the experiment, reversion to the usual attentional interference effect would be strong evidence that the modulation of RT by aversive pictures is the summation of attentional and motivational (activation of defensive response) effects.

## Methods

### Participants

Ninety volunteers (60 female) participated in the main RT experiment (aged between 18–29 years). Additionally, 123 volunteers (76 female) performed supplementary psychometric experiments consisting of characterizing emotional and neutral stimuli, i.e., to obtain complexity, valence, arousal and threat perception evaluations associated with each stimulus (see below). All participants were undergraduate students of the Federal Fluminense University, Niteroi, Brazil, who reported no history of neurological or psychiatric disorders and were not taking any medications that act on the central nervous system. All participants had normal or corrected vision. The local ethics committee approved the experimental protocol, and each participant gave written informed consent prior to participation.

### Apparatus and stimuli

Participants were tested in a sound-attenuated room under dim ambient light. A computer controlled stimulus timing and presentation and response collection. Stimulus presentation was programmed using E-Prime^®^ software (Psychology Software Tools Inc., Pittsburgh, PA). The participants’ heads were positioned on a head-and-chin rest situated 57 cm from the screen.

Twenty photos (10 threat and 10 neutral stimuli) were used. Pictures were either obtained from the World Wide Web or photographed by the authors, with the exception of one picture that was obtained from the International Affective Picture System (IAPS; Lang et al., 2005). Threat stimuli were divided in two sets: one set (*n* = 5) was composed of photographs with a person directing a firearm toward the observer (threat directed toward the observer), and the other set (*n* = 5) was composed of photographs in which a person was pointing a firearm away from the viewer (threat directed away from the observer).

The neutral stimuli (*n* = 10) were also divided into two sets and were composed of photos of people in everyday situations, neutral faces, and body parts. We attempted to match neutral stimuli with each threat stimuli set in terms of picture composition (e.g., number of faces, color content, number of body parts, number of people, etc.). Thus, for each threat stimuli set, there was a set of paired neutral stimuli. Another aspect that we tried to match between each set of threat and neutral stimuli was picture complexity. Matching was used as a control technique because a study by Bradley et al. (2007) suggested that some differences observed when recording neural responses to neutral and emotional pictures may be due to variations in picture complexity (clear figure-ground pictures × complex scenes depicting multiple objects) rather than the emotionality of each picture. In our study, we attempted to minimize this aspect by selecting only emotional and neutral stimuli that appeared to be of the same complexity level, i.e., clear figure-ground pictures. To ensure that our a priori selection was adequate, we asked an independent sample of 51 students (32 female) to rate picture complexity on a 1–9 scale (1: clear figure-ground, 9: complex scene) following the procedures described by Bradley et al. (2007). The results corroborated our a priori selection of pictures; the mean complexity rating of each picture in the set of threat and neutral stimuli was less than 3 (see Bradley et al., 2007).

Additionally, following the protocol developed by Lang et al. (1997), all images were assessed on a 1–9 scale in terms of valence (from negative to positive) and arousal (from low to high) by a separate group of 46 (28 female) graduate students using the paper-and-pencil version of the Self-Assessment Manikin (Bradley and Lang, 1994). The mean values obtained for valence and arousal rating sessions are described in Table 1.

**Table 1:** **Mean valence, arousal, reaction time and error rate for neutral and threat stimuli.**

	**Directed towards block**				**Directed away block**			
	**Threat**	**Threat**	**SD**	**Neutral**	**SD**	**Threat**	**SD**	**Neutral**	**SD**
**Valence**	3.24	0.22	5.33	0.40	2.65	0.46	4.76	0.38
**Arousal**	6.03	0.40	3.40	0.51	5.93	0.71	3.01	0.35
**Mean reaction time**	579	73	603	74	590	73	580	63
**Error rate(%)**	8.35	9.05	10.34	9.95	8.97	9.95	7.66	7.64

### Design and procedure

The experimental session was divided into two blocks. During each block, one set of emotional stimuli (threat directed toward the observer or threat directed away from the observer) and its paired neutral stimuli were presented three times. This yielded 15 neutral and 15 emotional trials per block. Therefore, the experimental session consisted of one block of photos with threat stimuli directed toward the observer and its matched neutral photos and another block of photos with threat stimuli directed away from the observer and its matched neutral photos. These blocks are henceforth referred to as “directed toward block” and “directed away block”, respectively. The order of neutral and threat images within a block was randomized, as was the order of the blocks between participants.

**Figure 1:**
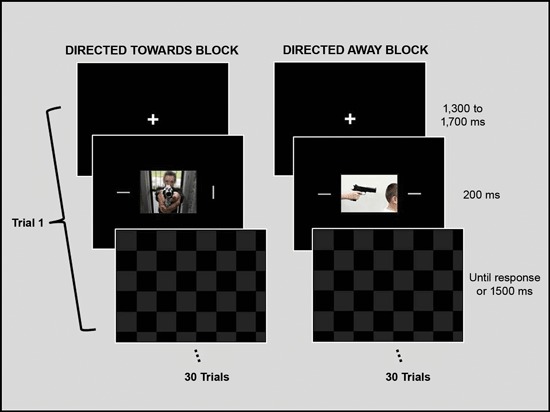
**Schematic representation of the experimental design.** The experimental session was divided into two blocks. During each block, one set of emotional stimuli (threat directed toward the observer or threat directed away from the observer) and its paired neutral stimuli were presented and repeated twice, totaling 15 neutral and 15 emotional stimuli per block. The order of neutral and threat images within a block was randomized, as was the order of the blocks between participants. Each trial began with a fixation cross that was presented for 1500 ms, which was followed by a central picture and two peripheral bars, which were presented for 200 ms. A checkerboard mask then appeared and remained on the screen until the participant gave a response or the maximum amount of allotted time (1500 ms) passed. Participants were instructed to ignore the task-irrelevant central images and to respond as fast and as accurately as possible regarding the orientation of the peripheral bars by indicating whether the orientation of the bars was the same. Stimuli are not drawn to scale.

The experimental design was similar to one used by Erthal et al. (2005). Each trial began with a fixation cross, which was displayed for 1500 (± 200) ms. Next, a central picture (9° × 12°) and two peripheral bars (0.3° × 3.0°) were presented for 200 ms. The bars were situated at 9° to the right and left of the center of the picture. A whole-screen black and grey checkerboard mask was then shown and remained on the screen until the participant responded or for a maximum of 1500 ms. Participants were instructed to ignore the task-irrelevant central image and to respond as fast and as accurately as possible to the peripheral bars, indicating whether their orientation was the same. Key presses (using the right or left index finger) corresponding to same/different orientation judgments were counterbalanced across participants. The angular difference of the bars in non-matching trials was 90°, and each block contained the same number of matching and non-matching trials. Each experimental block consisted of 15 trials with neutral photos and 15 trials with threat photos. There was a brief rest interval (2 to 3 min) between blocks. See Figure 1 for an illustration of the experimental design.

Participants performed a practice block prior to performing the two experimental blocks. An additional set of 10 neutral pictures of household objects from the IAPS (Lang et al., 2005) was selected for the practice block. During this training block, participants received feedback on the screen, which indicated anticipatory responses (RTs less than 100 ms), slow responses (RTs greater than 1500 ms), incorrect key responses and the RTs for correct trials. The training block was the only block in which feedback was given; it was not included in the analyses.

### Threat perception rating

As mentioned before, Blanchard et al. (2001) demonstrated that particular features of threat stimuli that are determinant for triggering a defensive strategy in animals appear to be fundamentally equal for humans. According to these authors, the type of human defensive behavior evoked by threat stimuli is determined by factors such as: the magnitude of the threat, the escapability of the situation, the distance between the threat and the participant and the presence of available hiding places. To evaluate whether the stimuli used in the present experiment varied in these features, we asked a different group of 26 volunteers (16 female) from the Federal Fluminense University to rate each set of stimuli. All pictures of the same set were presented in sequence, and each picture was presented for 3 sec. After viewing the stimuli, partipants rated each set using a Likert scale to quantify the images in the following dimensions:

1. magnitude of threat (1–9 scale, 9 represented the highest extent of threat)
2. distance between threat and subject (9 corresponded to the closest distance)
3. escapability or inescapability of threat (9 corresponded to the lowest escapability)
4. possibility of hiding from the threat (9 corresponded to the lowest possibility).

Participants were instructed to rate each set of stimuli according to their subjective feelings when viewing the stimuli. An additional set of threat pictures that was not used in the experiment (animal pictures) was used for training in this group of participants.

The ratings obtained for the four dimensions of the threat perception scale were summed. We then subtracted the ratings for the neutral set from those of its matched threat set to create a threat perception index per block. In other words, the ratings from the neutral stimuli presented in the direct toward block were subtracted from those of the threat directed toward the observer stimuli, and the ratings from the neutral stimuli presented in the directed away block were subtracted from the threat stimuli directed away from the observer. The threat perception indices for each block (directed toward and directed away blocks) were compared using a two-tailed paired *t*-test; the alpha level for statistical significance in this analysis was *P* < 0.05.

### Trauma history

A recent study by Purkis et al. (2011) demonstrated that the interference produced by emotional pictures was dependent on the relevance of these stimuli for each individual. In the present study, emotional stimuli used were pictures of guns (directed toward or directed away from the observer) and we wondered if participants who had been previously exposed to more types of violent crimes during their lives might react differently to these stimuli, especially when the gun is pointed toward the individual. To assess this issue, we asked the participants to complete the Trauma History Questionnaire (THQ; translated and adapted to Portuguese by (Fiszman et al., 2005) from the original (Green, 1996). The THQ is 23-item list of potentially traumatic events that address a range of events in three areas: crime-related events (e.g., robbery, mugging), general disaster and trauma (e.g., injury, disaster, witnessing death), and unwanted physical and sexual experiences. The THQ also contains an open-ended question for specifying any other extraordinarily stressful situations or events that were previously experienced. Fifty-seven participants completed the questionnaire after completing the RT task. For each event type, the respondent indicated whether he or she had experienced it during his or her lifetime. In this study, we summed the number of types of “violent crime” to which participants had been exposed. The THQ items that were considered as “violent crime” were selected according to the Luz et al. (2011) trauma categorization, which included any type of non-specific crime or act of violence (physical/violent assault, crime/violence victims, community/workplace/urban interpersonal violence, robbery, shooting, arson or aggression). Sexual assault, violence in war situations, domestic violence and child abuse were not included as “violent crime”. As mentioned before, we decided to specifically investigate violent crime because we hypothesized that participants with high numbers of this type of traumatic experience might react differently to threat stimuli. Increased violent crime exposure might determine an increase in the relevance of the threat stimuli and its influence, especially when the threat is a weapon directed toward the observer. In the analysis of the effect of violent crime-related trauma load, only participants who reported at least one violent crime experience were included (*n* = 48). After computing the total violent crime trauma score for each participant, we used a mean-split to separate respondents into two groups. Participants with a number of types of violent crime experiences below the mean (< 1.6) were classified as the low-trauma group, and those with a number of types of violent crime experiences above the mean were classified as the high-trauma group. Forty-six percent (*n* = 22) of the sample met the criteria for the high-trauma group.

### Reaction time data analysis

All anticipatory, slow or incorrect responses were excluded from further analyses. Four participants were excluded due to excessive errors (more than 50 % of the trials). The mean error ratings for each block are presented in Table 1. Outliers (*n* = 7) with mean RTs greater than three standard deviations from the sample mean were excluded from all analyses.

We calculated each participant’s mean RT for the directed toward the observer threat and directed away trials and their corresponding matched neutral trials. We then created an emotional modulation index for each block (the directed toward block stimuli and the directed away block stimuli) by subtracting the mean response time to targets in neutral trials from those of threat trials. Positive values of the emotional modulation index represent that participants were slower for threat stimuli than for neutral stimuli; negative values indicate that participants were faster for threat stimuli than for neutral stimuli. After calculating the emotional modulation index, we proceeded with the analysis in two steps. In the first step, emotional modulation scores for each block were compared using a two-way paired *t*-test to explore whether emotional modulation was different between blocks. In the second step, we tested whether the emotional modulation observed in each block was significantly different from zero by computing one-sample *t*-tests for each block.

To investigate whether the emotional modulation effect was influenced by violent crime trauma load, we performed a repeated-measures ANOVA with “block” (directed toward the observer block stimuli and no direct block stimuli) as the within-subjects factor and “trauma group” (high-trauma and low-trauma) as a between-groups factor. Planned comparisons were used to investigate critical contrasts between the high- and low-trauma groups in each block. For all analyses, the alpha level for statistical significance was *P* < 0.05.

## Results

### Threat perception ratings

**Figure 2:**
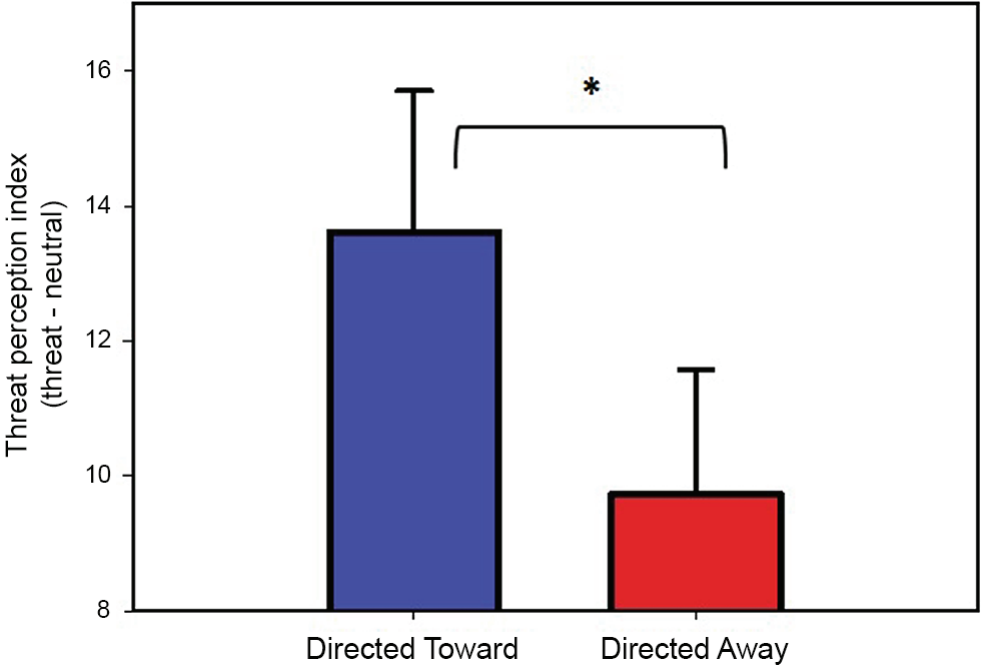
**Threat perception ratings.** Values represent the threat perception index (Threat-Neutral ratings) per block. Both bars represent the mean values, and error bars indicate the SEM. * Indicates a significant difference.

The threat perception index for each block (directed toward and directed away) was compared using a two-tailed paired *t*-test, which revealed that the threat index for the directed toward block (13.6) was significantly greater (*t*(25) = 2.61, *P* = 0.01) than the threat index for the directed away block (9.7). These data indicate that threat in the directed toward block was considered more intense, near and inescapable and that there was a reduced possibility of hiding. The results obtained from the threat perception scale are illustrated in Figure 2.

### Reaction time

**Figure 3:**
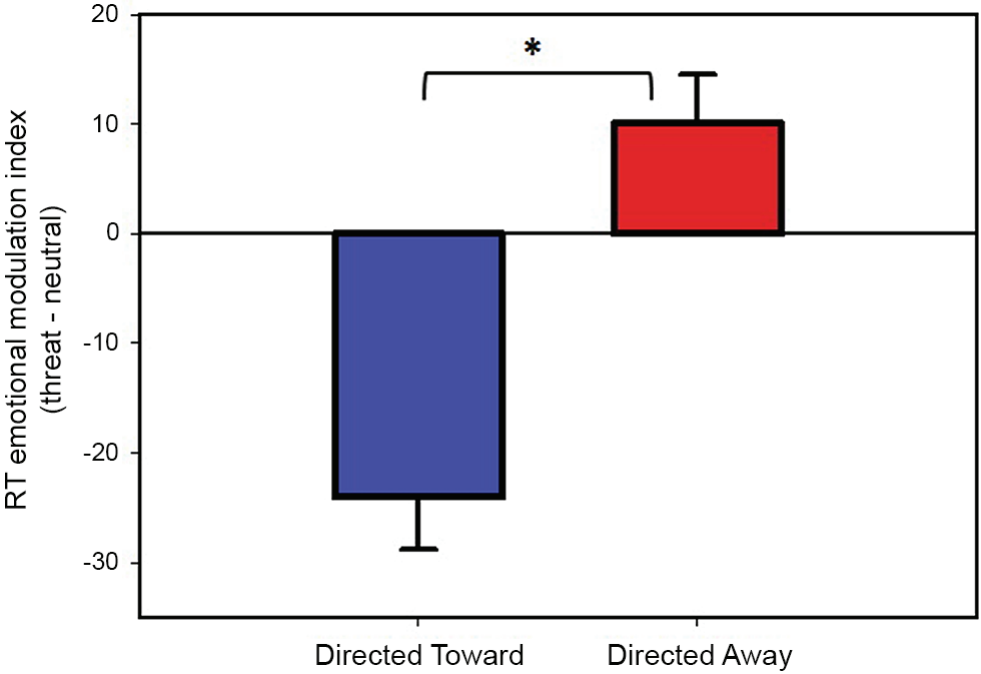
**Reaction time modulation by threat stimuli.** Values represent the threat emotional modulation index (Threat-Neutral RT) per block. Both bars represent mean values in ms, and error bars indicate the SEM. Positive values of the emotional modulation index indicate that participants were slower in responding when threat stimuli were present than when neutral stimuli were present; negative values indicate that participants were faster in responding when threat stimuli were present than when neutral stimuli were present. * Indicates a significant difference.

The modulation indices obtained during the directed toward and the directed away blocks are illustrated in Figure 3. We found a negative modulation index (−24 ms) in the directed toward block, which indicates that participants’ RTs were faster for threat than neutral stimuli. However, we measured a positive modulation index (10 ms) during the directed away block; this indicates that participants’ RTs were slower for threat stimuli than for neutral stimuli.

We compared the emotional modulation index obtained for each block to test whether the indices obtained were significantly different from each other. The results demonstrated that the emotional modulation index obtained during the directed toward block was significantly different (*t*(78) = 5.39, *P* < 0.001) from that obtained in the directed away block. If outliers are not excluded from the data the results are similar, with a negative modulation index (−21 ms) in the directed toward block and a positive modulation index (11 ms) during the directed away block and they differ significantly from each other (*t*(85) = 4.06, *P* < 0.001).

Next, we tested whether each emotional modulation index was different from zero using a one-sample *t*-test. The analysis revealed that both the directed toward and the directed away block modulation indices were significantly different from zero (*t* (78) = −5.02, *P* < 0.001); (*t*(78) = 2.29, *P* < 0.05), respectively. If outliers are not excluded from the data, the modulation index obtained for both blocks are significantly different from zero ((*t* (85) = −4.00, *P* < 0.001) and (*t*(85) = 2.05, *P* < 0.05), for the directed toward and directed away block respectively).

### Effect of violent crime trauma load

**Figure 4:**
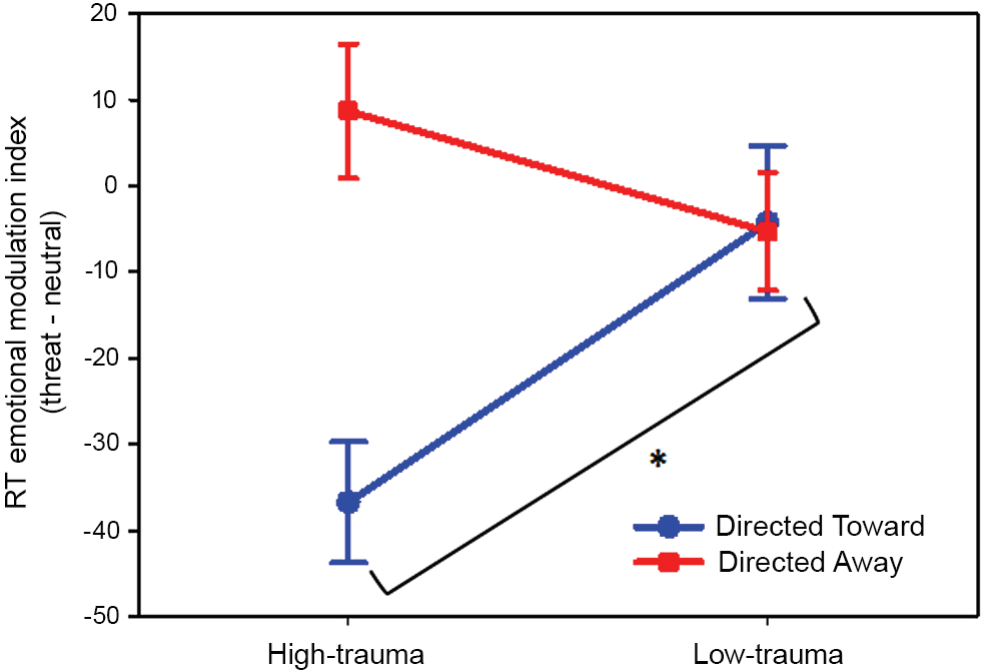
**Effect of violent crime trauma load on the emotional modulation produced by threat pictures.** Values represent the threat emotional modulation index per block. Participants were divided into high- and low-trauma groups. In all cases, the mean values are reported in ms and are plotted with error bars indicating the SEM. * Indicates a significant difference.

The analysis of variance revealed a significant main effect of block (*F*(1,46) = 11.78; *P* < 0.01) and, interestingly, an interaction effect between block and trauma group (*F*(1,46) = 12.95; *P* < 0.001). Planned comparisons revealed that the emotional index of the directed toward block differed significantly (*P* < 0.01) between the high- and low-trauma groups. For the directed away block, there was no significant difference between trauma groups (*P* = 0.18). These results are illustrated in Figure 4.

## Discussion

The results of this study revealed that presenting different threat stimuli produces varying types of RT modulation. A distractor threat stimuli directed toward the observer resulted in reduced RT in judging the orientation of peripheral bars when compared the same paradigm involving neutral distractors. However, threat distractors directed away from the observer increased RT in performing the same task when compared to neutral distractor stimuli. These opposing effects on RT may be interpreted as evidence that when presenting emotional pictures, factors other than attentional effects can modulate participant performance in behavioral tasks. Threat direction was manipulated as an attempt to activate various defensive responses. Our results suggest that this manipulation was successful: threat directed toward the observer was judged as more intense, near and inescapable and with less possibility of hiding. Directed threat stimuli likely activated the defense cascade more powerfully and prompted intense motor preparation, as evidenced by accelerated RTs. In contrast, threat stimuli directed away from the observers likely activated a less intense stage of the defense cascade, which more likely prompted immobility responses. In the latter case, modulation of the motor system favored increased RTs during threat trials compared to neutral trials. Finally, the relevance of the threat stimuli for each individual was a key component in determining the intensity of defensive reactions. Participants who had been exposed to a wider range of violent crimes were influenced more strongly by threat stimuli directed toward them than participants who had less exposure to violent crimes.

A large number of previously reported studies have explored the effects of unpleasant stimuli on behavior and have consistently reported that emotional stimuli produce interference effects when the stimuli are distractive to the task (Bradley et al., 1996; Hartikainen et al., 2000; Pessoa et al., 2002; Erthal et al., 2005; Pessoa, 2005; Yates et al., 2010; Hindi Attar and Müller, 2012). The most commonly cited explanation for this effect is that emotional stimuli connote an attentional competitive advantage, possibly mediated by the amygdala (Anderson and Phelps, 2001), which prioritizes emotional information processing (Pessoa et al., 2002). The reasoning underlying this interpretation is that interference during the simultaneous presentation of emotional and neutral items occurs because the emotional item is prioritized, which diverts brain resources away from processing neutral items, increasing RT.

However, in addition to the largely reported attentional effects, viewing unpleasant stimuli activates the defense system and prompts defensive reactions (Bradley et al., 2001; Azevedo et al., 2005). Azevedo et al. (2005) reported direct evidence that unpleasant pictures induce freezing-like responses in humans in the laboratory. Considering that emotional stimuli should prompt actions that are beneficial to the organism (Darwin, 1872), it is expected that processing emotional items should influence motor output to prepare individuals for action. In fact, many studies have used a combination of electromyography and transcranial magnetic stimulation to present evidence supporting the idea that activity of motor-related areas is modulated by emotional processing in humans (Oliveri et al., 2003; Baumgartner et al., 2007; Hajcak et al., 2007). Furthermore, neuroimaging studies using functional magnetic resonance imaging have reported that experimentally induced states of fear engage motor circuits (Anderson and Phelps, 2001; Butler et al., 2007). Recently, de Oliveira et al. (2012) demonstrated that the amplitude of the readiness potential, which is an electrophysiological marker of motor preparation, is modulated by emotion; these authors suggested that emotionally laden stimuli recruit a pre-set motor repertoire that is consistent with the valence of the stimuli. These studies lend support to the concept that emotional stimuli modulate motor output and our previous studies using aversive pictures are consistent with this idea (Pereira et al., 2004, 2006, 2010; Souza et al., 2012).

In the present study, when threat was directed away from the observer, increased RTs were observed. This interference effect is likely the result of both attentional and motivational effects. As discussed above, when emotional items are irrelevant to a given task, they compete for brain resources, causing increased RTs. Additionally, threat stimuli directed away from the observer were considered less intense, near and inescapable, and it was considered more possible to hide from them. Blanchard et al. (2001) considered these aspects of stimuli evaluation to be determinants of defensive behaviors. It is reasonable to expect that an immobility reaction was prompted during non-direct threat trials. In this case, modulation of the motor system would also favor an increase in RTs.

However, when threat stimuli were directed toward the observer, we noted decreased RTs in judging the orientation of peripheral bars compared to the RTs observed in the same paradigm involving neutral distracter stimuli. The participants considered direct threat stimuli to be more intense, near and inescapable, and it was considered less possible to hide from them. There is considerable evidence that threat direction modulates emotional response (Dimberg and Öhman, 1983; Dimberg, 1986; Hugdahl and Johnsen, 1989; Carlson et al., 2009; Grèzes et al., 2013), and, as suggested by Flykt et al. (2007), threat direction is readily associated with the concept of threat imminence. As previously described, the perception of increased threat imminence is one feature that determines defensive response strategies, which may change from freezing to defensive attack if escape is blocked (Blanchard et al., 2001). Thus, decreased RTs in direct threat trials may be interpreted as evidence that there is increased preparedness for action counteracting interference that may be produced by attentional effects. Similarly, Pichon et al. (2012) reported that threat signals were able to trigger responses in a subcorticocortical network related to motor vigilance and defensive behavior independently of attention. In their study, participants made judgments of color or emotion while watching short video clips of threatening scenarios. Threat stimuli prompted constant activity in the periaqueductal gray, hypothalamus, and premotor cortex that was task-independent. The authors argued that their results were consistent with the view that, at their core, emotions are action tendencies. Other evidence reported by Mobbs et al. (2007, 2010) describes how human defensive responses may be intensely activated by aversive stimuli. These authors demonstrated that motor-related areas were recruited when threat was extreme and closer to the observer, claiming that overt defensive reactions were likely prompted by the perception of increased threat imminence.

As a final point, we found that high- and low-trauma groups reacted differently when threat stimuli was directed toward the observer. Individuals who were exposed to more types of violent crime presented increased modulations due to threat stimuli. The relevance of the stimulus for the individual has been clearly demonstrated as very important in an interesting paper by Purkis et al. (2011). These authors showed that the magnitude of the interference produced in a visual search task varied according to the relevance of the stimuli for each individual. Similarly, studies using highly relevant emotional stimuli in phobic participants have suggested that the relevance of the stimuli determined the capacity of these stimuli to interfere with task performance (Okon-Singer et al., 2011). In addition, anxiety studies have showed that anxious participants exhibit greater interference resulting from threat-related stimuli and that the difficulty to filter threat-related distracters was exaggerated among anxious individuals (e.g., MacLeod et al., 1986; Mocaiber et al., 2009; Stout et al., 2013). A study of patients with post-traumatic stress disorder revealed increased defensive reactions to trauma-related stimuli (Volchan et al., 2011). In the present study, the emotional modulation effect produced by direct threat stimuli was influenced by the number of types of violent crime previously experienced by the participant. Threat stimuli used in the present study consisted of pictures of guns, and participants exposed to a wider range of violent crimes reacted differently to these stimuli, specifically when the stimulus consisted of a gun pointed toward the individual. We might suppose that repeated exposure to violent crime increased the relevance of this type of stimulus, resulting in enhanced reactivity to it. This result corroborates with Oliveira et al. (2013) suggestion that the relevance and the amount of influence produced by a distracter is highly modulated by differences between individuals.

In summary, when aversive stimuli are presented while participants perform a task, the influence that these stimuli have on motor output is an important determinant of the emotional modulation of behavior. In this study, the direction of the threat stimuli was the key factor manipulated to activate different defensive responses. The interference that is typically produced by attentional effects of distractive aversive stimuli was supplanted by threat stimuli directed toward the observer that triggered overt defensive actions (likely defensive attack). However, threat stimuli directed away from the observer, which activate the defense cascade less intensely than threat stimuli directed toward the observer, produced the typical interference effect (increased RT in non-direct threat trials compared to neutral trials). We may consider the reversion of reduced to accelerated RTs as threat direction varies as clear evidence that attentional and motivational effects interact to determine ongoing behavior. Additionally, the impact that threat stimuli have on participant behavior is dependent on the extent to which the stimuli are considered relevant to the individual. Finally, our data support the view that emotions are, in fact, action tendencies.

## Author contributions

Orlando Fernandes Jr., Eliane Volchan, Mirtes G. Pereira and Leticia de Oliveira conceptualized the study. All authors designed the study, Orlando Fernandes Jr. collected data and analyzed data. All authors contributed to data interpretation. Mirtes G. Pereira, Leticia de Oliveira and Orlando Fernandes Jr. wrote the paper. Mirtes G. Pereira, Orlando Fernandes Jr. and Rita de Cássia Soares Alves created the figures and table. Rita de Cássia Soares Alves inserted references. Mirtes G. Pereira and Leticia de Oliveira supervised the study. All authors contributed extensively to revising the paper.

## Conflict of interest statement

The authors declare that the research was conducted in the absence of any commercial or financial relationships that could be construed as a potential conflict of interest.

## References

[B1] Anderson A. K. Phelps E. A. (2001). Lesions of the human amygdala impair enhanced perception of emotionally salient events. Nature 411, 305–309 10.1038/35077083 11357132

[B2] Azevedo T. M. Volchan E. Imbiriba L. A. Rodrigues E. C. Oliveira J. M. Oliveira L. F. (2005). A freezing-like posture to pictures of mutilation. Psychophysiology 42, 255–260 10.1111/j.1469-8986.2005.00287.x 15943678

[B3] Baumgartner T. Willi M. Jäncke L. (2007). Modulation of corticospinal activity by strong emotions evoked by pictures and classical music: a transcranial magnetic stimulation study. Neuroreport 18, 261–265 10.1097/WNR.0b013e328012272e 17314668

[B4] Blanchard R. J. Blanchard D. C. (1989). Attack and defense in rodents as ethoexperimental models for the study of emotion. Prog. Neuropsychopharmacol. Biol. Psychiatry 13, 3–14 10.1016/0278-5846(89)90105-x 2694228

[B5] Blanchard R. J. Flannelly K. J. Blanchard D. C. (1986). Defensive behaviors of laboratory and wild Rattus norvegicus. J. Comp. Psychol. 100, 101–107 10.1037/0735-7036.100.2.101 3720282

[B6] Blanchard D. C. Hynd A. L. Minke K. A. Minemoto T. Blanchard R. J. (2001). Human defensive behaviors to threat scenarios show parallels to fear- and anxiety-related defense patterns of non-human mammals. Neurosci. Biobehav. Rev. 25, 761–770 Retrieved from http://www.ncbi.nlm.nih.gov/pubmed/11801300 10.1016/s0149-7634(01)00056-2 11801300

[B7] Bolles R. C. (1970). Species-specific defense reactions and avoidance learning. Psychol. Rev. 77, 32–48 10.1037/h0028589

[B8] Bradley M. M. Codispoti M. Cuthbert B. N. Lang P. J. (2001). Emotion and motivation I: defensive and appetitive reactions in picture processing. Emotion 1, 276–298 10.1037/1528-3542.1.3.276 12934687

[B9] Bradley M. M. Cuthbert B. N. Lang P. J. (1996). Picture media and emotion: effects of a sustained affective context. Psychophysiology 33, 662–670 Retrieved from http://www.ncbi.nlm.nih.gov/pubmed/8961788 10.1111/j.1469-8986.1996.tb02362.x 8961788

[B10] Bradley M. M. Hamby S. Löw A. Lang P. J. (2007). Brain potentials in perception: picture complexity and emotional arousal. Psychophysiology 44, 364–373 10.1111/j.1469-8986.2007.00520.x 17433095

[B11] Bradley M. M. Lang P. J. (1994). Measuring emotion: the self-assessment manikin and the semantic differential. J. Behav. Ther. Exp. Psychiatry 25, 49–59 Retrieved from http://www.ncbi.nlm.nih.gov/pubmed/7962581 10.1016/0005-7916(94)90063-9 7962581

[B12] Buodo G. Sarlo M. Palomba D. (2002). Attentional resources measured by reaction times highlight differences within pleasant and unpleasant, high arousing stimuli. Motiv. Emot. 26, 123–138 10.1023/A:1019886501965

[B13] Butler T. Pan H. Tuescher O. Engelien A. Goldstein M. Epstein J. (2007). Human fear-related motor neurocircuitry. Neuroscience 150, 1–7 10.1016/j.neuroscience.2007.09.048 17980493

[B14] Carlson J. M. Fee A. L. Reinke K. S. (2009). Backward masked snakes and guns modulate spatial attention. Evol. Psychol. 7, 534–544

[B15] Cuthbert B. N. Bradley M. M. Lang P. J. (1996). Probing picture perception: activation and emotion. Psychophysiology 33, 103–111 Retrieved from http://www.ncbi.nlm.nih.gov/pubmed/8851238 10.1111/j.1469-8986.1996.tb02114.x 8851238

[B16] Darwin C. (1872). The Expression of the Emotions in Man and Animals, ed Murray J. (London).

[B52] de Oliveira L. A. S. Imbiriba L. A. Russo M. M. Nogueira-Campos A. A. Rodrigues Ede C. Pereira M. G. (2012). Preparing to grasp emotionally laden stimuli. PloS One 7:e45235 10.1371/journal.pone.0045235 23024811 PMC3443242

[B17] Dimberg U. (1986). Facial expressions as excitatory and inhibitory stimuli for conditioned autonomic responses. Biol. Psychol. 22, 37–57 Retrieved from http://www.ncbi.nlm.nih.gov/pubmed/3697457 10.1016/0301-0511(86)90019-0 3697457

[B18] Dimberg U. Öhman A. (1983). The effects of directional facial cues on electrodermal conditioning to facial stimuli. Psychophysiology 20, 160–167 Retrieved from http://www.ncbi.nlm.nih.gov/pubmed/6844515 10.1111/j.1469-8986.1983.tb03282.x 6844515

[B19] Erthal F. S. de Oliveira L. Mocaiber I. Pereira M. G. Machado-Pinheiro W. Volchan E. (2005). Load-dependent modulation of affective picture processing. Cogn. Affect. Behav. Neurosci. 5, 388–395 Retrieved from http://www.ncbi.nlm.nih.gov/pubmed/16541809 10.3758/cabn.5.4.388 16541809

[B20] Fanselow M. S. (1991). “The midbrain periaqueductal gray as a coordinator of action in response to fear and anxiety,” in The Midbrain Periaqueductal Gray Matter, eds Depaulis A. Brandler R. (New York: Plenum Press), 151–173

[B21] Fanselow M. S. (1994). Neural organization of the defensive behavior system responsible for fear. Psychon. Bull. Rev. 1, 429–438 10.3758/bf03210947 24203551

[B22] Fiszman A. Cabizuca M. Lanfredi C. Figueira I. (2005). The cross-cultural adaptation to Portuguese of the Trauma history questionnaire to identify traumatic experiences. Rev. Bras. Psiquiatr. 27, 63–66 10.1590/s1516-44462005000100014 15867986

[B23] Flykt A. Esteves F. Ohman A. (2007). Skin conductance responses to masked conditioned stimuli: phylogenetic/ontogenetic factors versus direction of threat? Biol. Psychol. 74, 328–336 10.1016/j.biopsycho.2006.08.004 17049710

[B24] Green B. L. (1996). Psychometric Review of Trauma History Questionnaire (Self-Report), ed Stamm B. H. (Lutherville, MD: Sidran Press), 366–388

[B25] Grèzes J. Philip L. Chadwick M. Dezecache G. Soussignan R. Conty L. (2013). Self-relevance appraisal influences facial reactions to emotional body expressions. PloS One 8:e55885 10.1371/journal.pone.0055885 23405230 PMC3566069

[B26] Hajcak G. Molnar C. George M. S. Bolger K. Koola J. Nahas Z. (2007). Emotion facilitates action: a transcranial magnetic stimulation study of motor cortex excitability during picture viewing. Psychophysiology 44, 91–97 10.1111/j.1469-8986.2006.00487.x 17241144

[B27] Hamm A. O. Cuthbert B. N. Globisch J. Vaitl D. (1997). Fear and the startle reflex: blink modulation and autonomic response patterns in animal and mutilation fearful subjects. Psychophysiology 34, 97–107 Retrieved from http://www.ncbi.nlm.nih.gov/pubmed/9009813 10.1111/j.1469-8986.1997.tb02420.x 9009813

[B28] Hartikainen K. M. Ogawa K. H. Knight R. T. (2000). Transient interference of right hemispheric function due to automatic emotional processing. Neuropsychologia 38, 1576–1580 10.1016/s0028-3932(00)00072-5 11074080

[B29] Hindi Attar C. Müller M. M. (2012). Selective attention to task-irrelevant emotional distractors is unaffected by the perceptual load associated with a foreground task. PloS One 7:e37186 10.1371/journal.pone.0037186 22649513 PMC3359362

[B30] Hugdahl K. Johnsen B. H. (1989). Preparedness and electrodermal fear-conditioning: ontogenetic vs phylogenetic explanations. Behav. Res. Ther. 27, 269–278 Retrieved from http://www.ncbi.nlm.nih.gov/pubmed/2730508 10.1016/0005-7967(89)90046-6 2730508

[B31] Ishai A. Pessoa L. Bikle P. C. Ungerleider L. G. (2004). Repetition suppression of faces is modulated by emotion. Proc. Natl. Acad. Sci. U S A 101, 9827–9832 10.1073/pnas.0403559101 15210952 PMC470759

[B32] Kalin N. H. (1993). The neurobiology of fear. Sci. Am. 268, 94–101 8386852 10.1038/scientificamerican0593-94

[B33] Keil A. Bradley M. M. Ihssen N. Heim S. Vila J. Guerra P. (2010). Defensive engagement and perceptual enhancement. Neuropsychologia 48, 3580–3584 10.1016/j.neuropsychologia.2010.08.007 20713075 PMC2949445

[B34] Klorman R. Weissberg R. P. Wiesenfeld A. R. (1977). Individual differences in fear and autonomic reactions to affective stimulation. Psychophysiology 14, 45–51 Retrieved from http://www.ncbi.nlm.nih.gov/pubmed/834802 10.1111/j.1469-8986.1977.tb01154.x 834802

[B35] Lang P. J. Bradley M. M. Cuthbert B. N. (1997). “Motivated attention: affect, activation and action,” in Attention and Orienting: Sensory and Motivational Processes, eds Lang P. J. Simons R. F. Balaban M. T. (Mahwah, NJ: Lawrence Erlbaum Associates), 97–135

[B36] Lang P. J. Bradley M. M. Cuthbert B. N. (2005). International Affective Picture System (IAPS): Affective Ratings of Pictures and Instruction Manual. Technical Report A-6. Gainesville, FL: University of Florida

[B37] Lang P. J. Davis M. Ohman A. (2000). Fear and anxiety: animal models and human cognitive psychophysiology. J. Affect. Disord. 61, 137–159 Retrieved from http://www.ncbi.nlm.nih.gov/pubmed/11163418 10.1016/s0165-0327(00)00343-8 11163418

[B38] Luz M. P. Mendlowicz M. Marques-Portella C. Gleiser S. Berger W. Neylan T. C. (2011). PTSD criterion A1 events: a literature-based categorization. J. Trauma Stress 24, 243–251 10.1002/jts.20633 21547956

[B39] MacLeod C. Mathews A. Tata P. (1986). Attentional bias in emotional disorders. J. Abnorm. Psychol. 95, 15–20 Retrieved from http://www.ncbi.nlm.nih.gov/pubmed/3700842 10.1037/0021-843x.95.1.15 3700842

[B40] Marks I. (1987). Fears, Phobias and Rituals: Panic, Anxiety, and Their Disorders. New York: Oxford University Press

[B41] Menezes C. Pereira M. G. Bizarro L. (2012). Sitting and silent meditation as a strategy to study emotion regulation. Psychol. Neurosci. 5, 27–36 10.3922/j.psns.2012.1.05

[B42] Mobbs D. Petrovic P. Marchant J. L. Hassabis D. Weiskopf N. Seymour B. (2007). When fear is near: threat imminence elicits prefrontal-periaqueductal gray shifts in humans. Science 317, 1079–1083 10.1126/science.1144298 17717184 PMC2648508

[B43] Mobbs D. Yu R. Rowe J. B. Eich H. FeldmanHall O. Dalgleish T. (2010). Neural activity associated with monitoring the oscillating threat value of a tarantula. Proc. Natl. Acad. Sci. U S A 107, 20582–20586 10.1073/pnas.1009076107 21059963 PMC2996708

[B44] Mocaiber I. Perakakis P. Pereira M. G. Pinheiro W. M. Volchan E. de Oliveira L. (2011). Stimulus appraisal modulates cardiac reactivity to briefly presented mutilation pictures. Int. J. Psychophysiol. 81, 299–304 10.1016/j.ijpsycho.2011.07.014 21820017

[B45] Mocaiber I. Pereira M. G. Erthal F. S. Machado-Pinheiro W. David I. A. Cagy M. (2010). Fact or fiction? An event-related potential study of implicit emotion regulation. Neurosci. Lett. 476, 84–88 10.1016/j.neulet.2010.04.008 20385204

[B46] Mocaiber I. Pereira M. G. Erthal F. Figueira I. Pinheiro W. Cagy M. (2009). Regulation of negative emotions in high trait anxious individuals: an ERP study. Psychol. Neurosci. 2, 211–217 10.3922/j.psns.2009.2.014

[B47] Mocaiber I. Sanchez T. A. Pereira M. G. Erthal F. S. Joffily M. Araujo D. B. (2011). Antecedent descriptions change brain reactivity to emotional stimuli: a functional magnetic resonance imaging study of an extrinsic and incidental reappraisal strategy. Neuroscience 193, 241–248 10.1016/j.neuroscience.2011.07.003 21782901

[B48] Ochsner K. N. Gross J. J. (2005). The cognitive control of emotion. Trends Cogn. Sci. 9, 242–249 10.1016/j.tics.2005.03.010 15866151

[B49] Öhman A. Lundqvist D. Esteves F. (2001). The face in the crowd revisited: a threat advantage with schematic stimuli. J. Pers. Soc. Psychol. 80, 381–396 Retrieved from http://www.ncbi.nlm.nih.gov/pubmed/11300573 10.1037/0022-3514.80.3.381 11300573

[B50] Okon-Singer H. Alyagon U. Kofman O. Tzelgov J. Henik A. (2011). Fear-related pictures deteriorate the performance of university students with high fear of snakes or spiders. Stress 14, 185–193 10.3109/10253890.2010.527401 21034301

[B51] Oliveri M. Babiloni C. Filippi M. M. Caltagirone C. Babiloni F. Cicinelli P. (2003). Influence of the supplementary motor area on primary motor cortex excitability during movements triggered by neutral or emotionally unpleasant visual cues. Exp. Brain Res. 149, 214–221 10.1007/s00221-002-1346-8 12610690

[B53] Oliveira L. A. S. Oliveira L. Joffily M. Pereira-Junior P. P. Lang P. J. Pereira M. G. (2009). Autonomic reactions to mutilation pictures: positive affect facilitates safety signal processing. Psychophysiology 46, 870–873 10.1111/j.1469-8986.2009.00812.x 19386048

[B54] Oliveira L. Mocaiber I. David I. A. Erthal F. Volchan E. Pereira M. G. (2013). Emotion and attention interaction: a trade-off between stimuli relevance, motivation and individual differences. Front. Hum. Neurosci. 7:364 10.3389/fnhum.2013.00364 23874284 PMC3709171

[B55] Pereira M. G. de Oliveira L. Erthal F. S. Joffily M. Mocaiber I. F. Volchan E. (2010). Emotion affects action: midcingulate cortex as a pivotal node of interaction between negative emotion and motor signals. Cogn. Affect. Behav. Neurosci. 10, 94–106 10.3758/cabn.10.1.94 20233958 PMC2875262

[B56] Pereira M. G. Volchan E. de Souza G. G. L. Oliveira L. Campagnoli R. R. Pinheiro W. M. (2006). Sustained and transient modulation of performance induced by emotional picture viewing. Emotion 6, 622–634 10.1037/1528-3542.6.4.622 17144753 PMC2376807

[B57] Pereira M. G. Volchan E. Oliveira L. Machado-Pinheiro W. Rodrigues J. A. Nepomuceno F. V. P. (2004). Behavioral modulation by mutilation pictures in women. Braz. J. Med. Biol. Res. 37, 353–362 Retrieved from http://www.ncbi.nlm.nih.gov/pubmed/15060703 10.1590/s0100-879x2004000300011 15060703

[B58] Pessoa L. (2005). To what extent are emotional visual stimuli processed without attention and awareness? Curr. Opin. Neurobiol. 15, 188–196 10.1016/j.conb.2005.03.002 15831401

[B59] Pessoa L. (2010). Attention and emotion. Scholarpedia 5:6314 10.4249/scholarpedia.6314

[B60] Pessoa L. Kastner S. Ungerleider L. G. (2002). Attentional control of the processing of neutral and emotional stimuli. Cogn. Brain Res. 15, 31–45 10.1016/s0926-6410(02)00214-8 12433381

[B61] Pessoa L. Padmala S. Morland T. (2005). Fate of unattended fearful faces in the amygdala is determined by both attentional resources and cognitive modulation. Neuroimage 28, 249–255 10.1016/j.neuroimage.2005.05.048 15993624 PMC2427145

[B62] Pichon S. de Gelder B. Grèzes J. (2012). Threat prompts defensive brain responses independently of attentional control. Cereb. Cortex 22, 274–285 10.1093/cercor/bhr060 21666127

[B63] Pourtois G. Schwartz S. Seghier M. L. Lazeyras F. Vuilleumier P. (2006). Neural systems for orienting attention to the location of threat signals: an event-related fMRI study. Neuroimage 31, 920–933 10.1016/j.neuroimage.2005.12.034 16487729

[B64] Purkis H. M. Lester K. J. Field A. P. (2011). But what about the Empress of Racnoss? The allocation of attention to spiders and doctor who in a visual search task is predicted by fear and expertise. Emotion 11, 1484–1488 10.1037/a0024415 21707142

[B65] Ratner S. C. (1967). “Comparative aspects of hypnosis,” in Handbook of Clinical and Experimental Hypnosis, ed Gordon J. E. (New York: Macmillan), 550–587

[B66] Souza G. G. L. Mendonça-de-Souza A. C. F. Barros E. M. Coutinho E. F. S. Oliveira L. Mendlowicz M. V. (2007). Resilience and vagal tone predict cardiac recovery from acute social stress. Stress 10, 368–374 10.1080/10253890701419886 17853065

[B67] Souza G. G. L. Pereira M. G. Vila J. Oliveira L. Volchan E. (2012). Affiliative stimuli as primers to prosocial predispositions. Span. J. Psychol. 15, 237–243 Retrieved from http://www.ncbi.nlm.nih.gov/pubmed/22379713 10.5209/rev_SJOP.2012.v15.n1.37315 22379713

[B72] Stout D. Shackman A. Larson C. (2013). Failure to filter: anxious individuals show inefficient gating of threat from working memory. Front. Hum. Neurosci. 7:58 10.3389/fnhum.2013.00058 23459454 PMC3586709

[B68] Tipples J. Sharma D. (2000). Orienting to exogenous cues and attentional bias to affective pictures reflect separate processes. Br. J. Psychol. 91, 87–97 Retrieved from http://www.ncbi.nlm.nih.gov/pubmed/10717773 10.1348/000712600161691 10717773

[B69] Volchan E. Souza G. G. L. Franklin C. M. Norte C. E. Rocha-Rego V. Oliveira J. M. (2011). Is there tonic immobility in humans? Biological evidence from victims of traumatic stress. Biol. Psychol. 88, 13–19 10.1016/j.biopsycho.2011.06.002 21693167

[B70] Wendt J. Lotze M. Weike A. I. Hosten N. Hamm A. O. (2008). Brain activation and defensive response mobilization during sustained exposure to phobia-related and other affective pictures in spider phobia. Psychophysiology 45, 205–215 10.1111/j.1469-8986.2007.00620.x 17995911

[B71] Yates A. Ashwin C. Fox E. (2010). Does emotion processing require attention? The effects of fear conditioning and perceptual load. Emotion 10, 822–830 10.1037/a0020325 21058839 PMC3491873

